# Retraction: Apigenin inhibits epithelial-mesenchymal transition of human colon cancer cells through NF-κB/Snail signaling pathway

**DOI:** 10.1042/BSR20190452_RET

**Published:** 2026-02-04

**Authors:** Jiafeng Tong, Ying Shen, Zhenghua Zhang, Ye Hu, Xu Zhang, Li Han

**Keywords:** apigenin, epithelial-mesenchymal transition (EMT), colon cancer cells, Snail

This article is being retracted from *Bioscience Reports* at the request of the Editor-in-Chief and the Editorial Board. This follows the receipt of a notification from a reader, alerting the Editorial Board to multiple similarities detected within this manuscript and between those published by other authors.

Specifically, these similarities include:

- In Figure 2A, the Occludin 200 mg and 300 mg panels appear to be highly similar to the Vimentin Control panel.
- A region of the Figure 4E 10 μM panel appears similar to a region of the 4F 10 μM panel.
- Similarities between the Figure 2A N-cadherin control panel and the Figure 6A N-cadherin control panel of Qin et al. 2016 (10.18632/oncotarget.9404)
- Similarities between the Figure 2A E-cadherin 300 mg/kg and the Figure 6A Vinmentin Control panel of Qin et al. 2016
- Similarities between Figure 2A N-cadherin 200 mg/kg and the Figure 6A E-cadherin 200 mg/kg of Qin et al. 2016
- Similarities between Figure 2A E-cadherin 200 mg/kg and the Figure 6A Occludin 200 mg/kg of Qin et al. 2016

The authors have been contacted with regards to the retraction and have not responded to the Journal's queries or the concerns raised. Given the extent of the issues raised, the Editorial Board stand by the decision to retract the article.

